# Chest Pain With Apical Diverticulum in the Absence of Coronary Disease: Case Report and Review of the Literature

**DOI:** 10.14740/cr442e

**Published:** 2015-12-16

**Authors:** Sushee Gadde, Bassam Omar

**Affiliations:** a Division of Cardiology, University of South Alabama, Mobile, AL 36617, USA

**Keywords:** Diverticulum, Aneurysm, Congenital anomaly

## Abstract

Aneurysmal dilatation of segment of the left ventricle in the absence of coronary disease has been reported and termed diverticulum, which appears to be a congenital anomaly. A 56-year-old white female was admitted to our hospital with chest pain that has been intermittent over the past 1 month. The pain was described as exertional, substernal and pressure-like in quality, radiating to left arm and jaw, and lasting approximately 30 minutes each episode; it was associated with shortness of breath. She has had approximately 10 such episodes in the past 1 month. The patient denied any dizziness, palpitations, syncope, orthopnea or paroxysmal nocturnal dyspnea (PND). She has had a history of hypertension for many years, however has not been compliant with her medications for the past 6 months. On admission, vital signs revealed blood pressure of 185/100 mm Hg, and regular heart rate of 94 beats per minute. Physical examination revealed a normal body habitus. Cardiac examination revealed no murmurs or extra cardiac sounds on auscultation. The pulmonary and abdomen examinations were unremarkable. The chest radiograph was normal. The electrocardiogram showed sinus rhythm, with borderline prolongation of the QT interval. The laboratory test results, including cardiac enzymes, were normal. Transthoracic echocardiography (TTE) revealed normal left ventricular systolic function, with localized dyskinesis of the apex. No significant valvular abnormalities were identified. Coronary angiography revealed angiographically normal coronary arteries; left ventriculography showed abnormal apical “filling defect” consistent with an aneurysm. A repeat echocardiogram using Definity contrast revealed left ventricular apical diverticulum with hypertrabeculation. The patient was placed on antihypertensive medications with resolution of her chest pain, and was able to ambulate comfortably. The patient was counseled thoroughly on the importance of compliance with her medications. This case describes an apical left ventricular diverticulum found incidentally and demonstrated on contrast echocardiography in a patient with chest pain.

## Introduction

A ventricular diverticulum is an outpouching of the left or right ventricle which may be found at various levels and can be isolated, or associated with other congenital anomalies. The clinical significance of this rare finding is yet to be delineated, although associations with various arrhythmic, embolic and rupture risks have been described. We present in this report an interesting case of a left ventricular apical diverticulum discovered incidentally during work-up of chest pain, in the absence of coronary artery disease, followed by review of pertinent literature.

## Case Report

**Figure 1:**
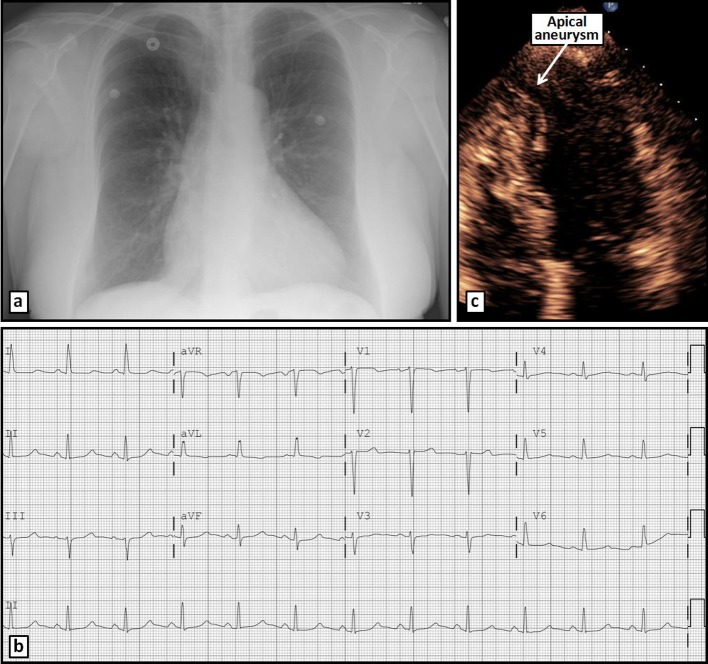
Chest radiograph (a) and ECG (b) on admission without significant abnormalities. Apical 4-chamber TTE view (c) reveals an apical aneurysm.

**Figure 2:**
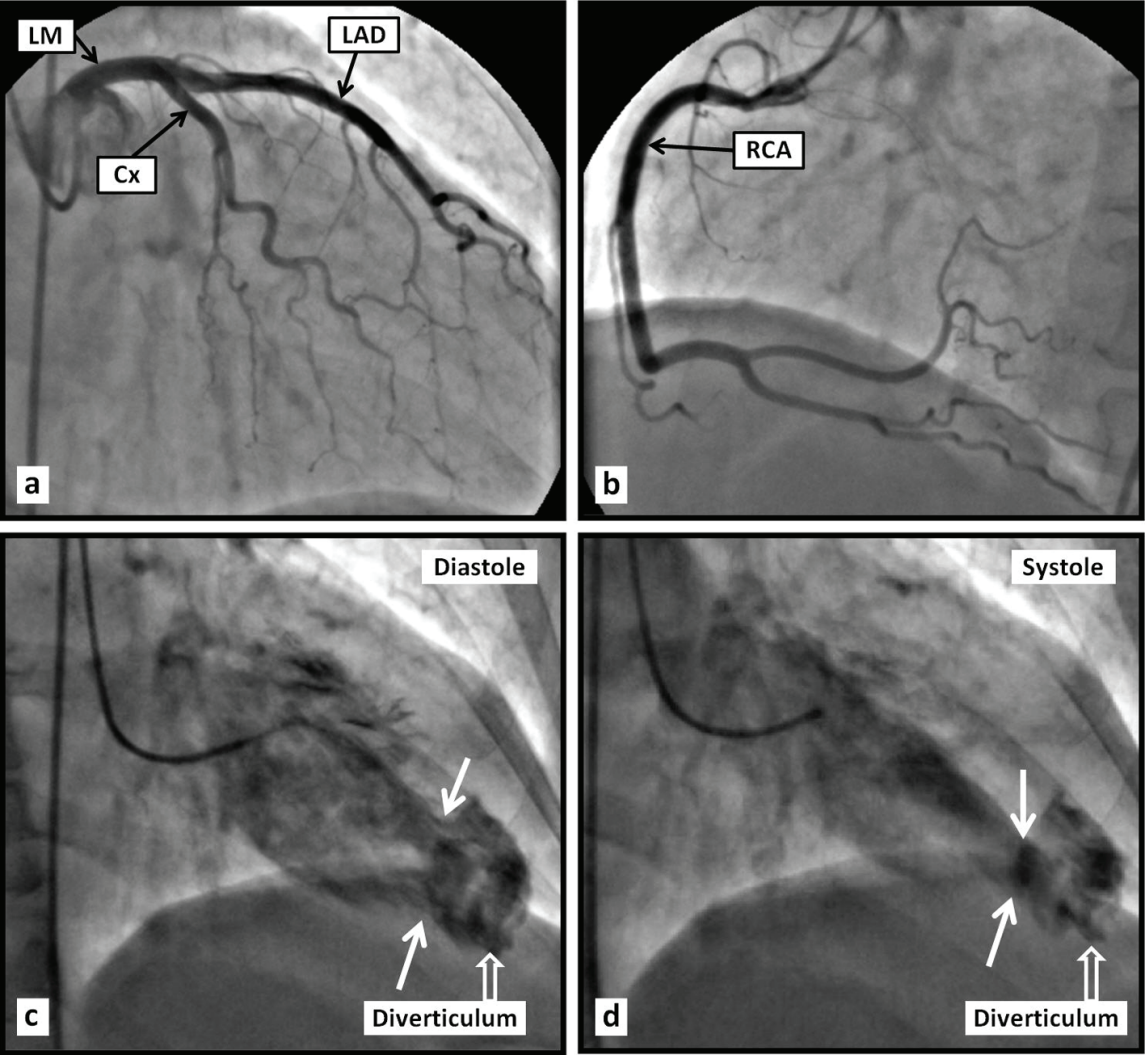
Coronary angiogram (a, b) reveals angiographically normal coronary arteries. Left ventriculography (c, d) reveals an apical aneurismal outpouching.

**Figure 3:**
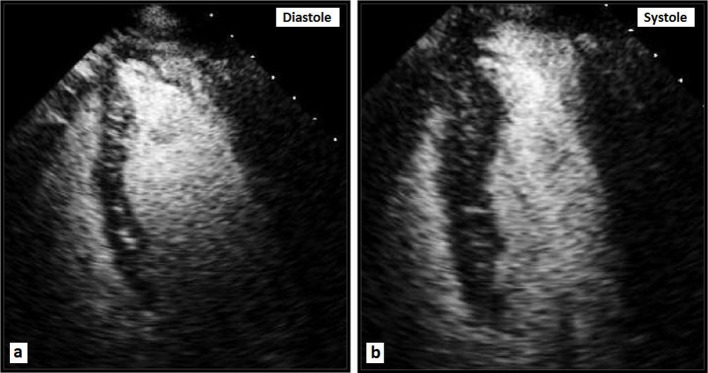
Apical four-chamber TTE views with Definity contrast demonstrating a large apical diverticulum.

A 56-year-old white female was admitted to our hospital with chest pain that has been intermittent over the past 1 month. The pain was described as exertional, substernal and pressure-like in quality, radiating to left arm and jaw, and lasting approximately 30 min each episode; it was associated with shortness of breath. She has had approximately 10 such episodes in the past 1 month. The patient denied any dizziness, palpitations, syncope, orthopnea or PND. She has had a history of hypertension for many years, however has not been compliant with her medications for the past 6 months. On admission, vital signs revealed blood pressure of 185/100 mm Hg, and regular heart rate of 94 beats per minute. Physical examination revealed a normal body habitus. Cardiac examination revealed no murmurs or extra cardiac sounds on auscultation. The pulmonary and abdomen examinations were unremarkable. The chest radiograph (Fig. 1a) was normal. The electrocardiogram (Fig. 1b) showed sinus rhythm, with borderline prolongation of the QT interval. The laboratory test results, including cardiac enzymes, were normal. Transthoracic echocardiography (TTE) revealed normal left ventricular systolic function, with localized dyskinesis of the apex (Fig. 1c). No significant valvular abnormalities were identified. Coronary angiography revealed angiographically normal coronary arteries (Fig. 2a, b); left ventriculography showed abnormal apical “filling defect” consistent with an aneurysm (Fig. 2c, d). A repeat echocardiogram using Definity contrast revealed left ventricular apical diverticulum with hypertrabeculation (Fig. 3a, b). The patient was placed on antihypertensive medications with resolution of her chest pain, and was able to ambulate comfortably. The patient was counseled thoroughly on the importance of compliance with her medications.

## Discussion

Ventricular diverticula are rare ventricular outpouchings distinct from aneurysms or pseudoaneurysms by the absence of congruent coronary disease. The true prevalence of left ventricular diverticulum remains unknown. Ohlow [1] claimed that there have been 411 reported cases of congenital left ventricular aneurysm or diverticulum since it was first described in 1816, 70% of which are associated with other cardiac, vascular or thoraco-abdominal abnormalities. Although most cases are clinically silent, some have been associated with systemic embolization, heart failure, valvular regurgitation, ventricular wall rupture, ventricular tachycardia and sudden cardiac death.

Nakazono et al [2] reported the incidence of ventricular diverticula on 256-slice multidetector computed tomography angiography (MDCTA) to be 3.4% on the left side and 0.6% on the right side. This seems greater than previously reported likely due to better detection techniques. Diverticula were predominantly found in the mid-inferoseptal or mid-anteroseptal left ventricular wall.

Srichai et al [3] found the prevalence of left ventricular diverticulum to be 2.2%, with no case of right ventricular diverticulum, on cardiac MDCTA studies performed for various suspected coronary artery abnormalities. They distinguished a diverticulum from an aneurysm by the presence of myocardial tissue, rather than fibrous tissue, around the ventricular protrusion.

Left ventricular diverticulum is often detected in childhood as part of a pentalogy, as described by Cantrell and colleagues [4] including congenital defects involving the abdominal wall, sternum, diaphragm, pericardium, and heart, with occasional associated limb malformations [5]. Cases of left ventricular diverticulum with partial Cantrell’s syndrome have also been described [6]. However, 30% of ventricular diverticula are isolated findings [7].

The presentation, diagnostic modalities and treatment of ventricular diverticulum have varied greatly in the literature, and appear to depend on the extent of involvement and symptoms [8]. Yazici et al [9] reported an apical muscular left ventricular diverticulum in a 65-year-old female with progressive dyspnea and palpitation, found to have atrial fibrillation with rapid ventricular response. She did well with medical management. Fatih et al [10] reported another case of incidental left ventricular apical diverticulum found on MDCT performed for atypical chest pain in a 47-year-old male. Similarly, Isilak et al [11] reported a 55-year-old male with atypical chest pain who had an incidental apical left ventricular diverticulum on echocardiography; he was treated medically.

Congenital left ventricular aneurysm, which is characterized by a wide connection to the ventricle, fibrosis with high signal on T2-weighted cardiac magnetic resonance imaging, and absence of other heart or midline thoraco-abdominal defects, can be rarely seen and may be confused with diverticula [12]. These appear to be associated with worse adverse outcomes compared with left ventricular diverticula.

Although the presence of concomitant coronary disease renders the differentiation of a left ventricular diverticulum from a left ventricular aneurysm difficult, several reports have been published describing the characterization of a diverticulum in the setting of coronary disease. Chang et al [13] reported a 76-year-old male with situs inversus who was diagnosed with a small basal left ventricular diverticulum on MDCT and angiography performed for chest pain evaluation. Coronary angiography revealed severe (95%) left anterior descending (LAD) and moderate (50%) middle left circumflex coronary artery obstructions. Keating et al [14] described a muscular diverticulum by CMR, which demonstrated prominent trabeculation without thrombus or scar on delayed enhancement images in a patient treated for acute coronary syndrome with distal RCA stent. Later, the patient underwent elective surgical repair of the diverticulum and coronary bypass of the LAD.

Although a left ventricular diverticulum is often diagnosed incidentally, serious associations with ventricular arrhythmia have been reported. Pitol et al [15] reported a 56-year-old female with normal coronary arteries who required cardioversion for a hemodynamically unstable monomorphic ventricular tachycardia (VT). She was found to have a posterobasal LV diverticulum, and underwent successful surgical resection. An earlier case was reported by Shen et al [16] in a 24-year-old male with recurrent monomorphic VT caused by an inferobasal diverticulum and resistant to many antiarrhythmics, which was successfully treated by surgical resection. Speechly-Dick et al [17] reported a 43-year-old female who was defibrillated from ventricular fibrillation; she was found to have normal coronary arteries and a diverticulum of the inferior and anterior walls.

Right ventricular (RV) diverticula are rare and discovered incidentally. Sattiraju et al [18] reported an incidental RV inferolateral diverticulum in a 57-year-old man on CT of the chest performed for further work-up of pneumonia, which was later confirmed with cardiac magnetic resonance (CMR). Lee et al [19] reported a 56-year-old woman with normal coronary arteries and two incidentally found diverticula along the inferoseptal wall of the RV on MDCT performed for chest pains.

### Conclusion

Cardiac right and left diverticula are rare, and are associated with different characteristic that have been summarized in Table 1 [20, 21]. Although in adults, they are often found incidentally, rare cases have been associated with significant complications which may necessitate aggressive medical treatment versus surgical resection of the diverticulum. Vigilance is called for when confronted with such a finding with regard to the potential associations and consequences, so that therapy can be tailored appropriately.

**Table 1:** Summary of the Characteristics of Fibrous Versus Muscular Diverticula

Characteristic	Fibrous	Muscular
Age	Adults	Children
Race	Predominately African Americans	No predilection
Prevalence	Not common	More Frequent
Histopathology	Mostly fibrous	Mostly muscular
Wall motion	Non-contractile, paradoxical motion	Contractile, synchronous motion
Segments affected	LV apical or subvalvular	LV apical; rarely RV
Complications reported	Aortic or mitral regurgitation; systemic embolism. Rupture. Arrhythmia	Rare complications
Associated abnormalities	No midline or congenital cardiac defects	Frequent midline and congenital cardiac defects
Angiography findings	No volume change during cardiac cycle	Volume reduction in systole and increase in diastole
MDCT findings	No volume change during cardiac cycle; fibrous tissue on diverticulum wall	Synchronous contraction with LV; myocardial tissue on diverticulum wall
CMR findings	Thin, fibrous wall; no volume change during cardiac cycle; no necrosis or fibrous tissue on delayed enhancement	Thin, contractile wall; no necrosis or fibrous tissue on delayed enhancement images
